# Photoimmunotherapy of Gastric Cancer Peritoneal Carcinomatosis in a Mouse Model

**DOI:** 10.1371/journal.pone.0113276

**Published:** 2014-11-17

**Authors:** Kazuhide Sato, Peter L. Choyke, Hisataka Kobayashi

**Affiliations:** Molecular Imaging Program, Center for Cancer Research, National Cancer Institute, Bethesda, Maryland 20892, United States of America; Columbia University, United States of America

## Abstract

Photoimmunotherapy (PIT) is a new cancer treatment that combines the specificity of antibodies for targeting tumors with the toxicity induced by photosensitizers after exposure to near infrared (NIR) light. We performed PIT in a model of disseminated gastric cancer peritoneal carcinomatosis and monitored efficacy with *in vivo* GFP fluorescence imaging. *In vitro* and *in vivo* experiments were conducted with a HER2-expressing, GFP-expressing, gastric cancer cell line (N87-GFP). A conjugate comprised of a photosensitizer, IR-700, conjugated to trastuzumab (tra-IR700), followed by NIR light was used for PIT. *In vitro* PIT was evaluated by measuring cytotoxicity with dead staining and a decrease in GFP fluorescence. *In vivo* PIT was evaluated in a disseminated peritoneal carcinomatosis model and a flank xenograft using tumor volume measurements and GFP fluorescence intensity. *In vivo* anti-tumor effects of PIT were confirmed by significant reductions in tumor volume (at day 15, p<0.0001 vs. control) and GFP fluorescence intensity (flank model: at day 3, PIT treated vs. control p<0.01 and peritoneal disseminated model: at day 3 PIT treated vs. control, p<0.05). Cytotoxic effects *in vitro* were shown to be dependent on the light dose and caused necrotic cell rupture leading to GFP release and a decrease in fluorescence intensity *in vitro.* Thus, loss of GFP fluorescence served as a useful biomarker of cell necrosis after PIT.

## Introduction

Gastric carcinoma causes more than 740,000 cancer-related deaths per year worldwide especially in Asia [1]–[3]. The majority of gastric cancer patients present with locally advanced, recurrent or metastatic disease precluding curative surgery that is mostly managed by non-curative therapy [1]. Peritoneal carcinomatosis and liver metastasis are common life-threatening manifestations of advanced stage gastric cancer [1], [4]. Peritoneal carcinomatosis also occurs in ovarian, appendiceal, colon, pancreas and gastric cancers. Prognosis is universally poor and local treatments are invariably unsuccessful with high recurrence rates and morbidity associated with ascites and bowel obstruction. Systemic therapy is also usually unsuccessful. Thus, new methods of treating peritoneal carcinomatosis are needed.

Photoimmunotherapy (PIT) is a new target-cell specific cancer treatment that employs an antibody photosensitizer conjugate (APSC) followed by near infrared (NIR) light exposure. An APSC consists of a cancer cell-specific monoclonal antibody (mAb) and a photosensitizer, IR700, which is a silica-phthalocyanine derivative covalently conjugated to the antibody. The APSC binds target molecules on the cell membrane and then induces nearly immediate cell necrosis after exposure to NIR light at 690 nm. *In vitro* studies have shown PIT to be highly cell-specific, therefore, non-expressing cells immediately adjacent to targeted cells show no toxic effects [5]. Cells treated with PIT undergo rapid volume expansion leading to rupture of the cell membrane, extrusion of cell contents into the extracellular space, and irreversible necrosis [6]–[8]. Our results and others demonstrate that cytotoxicity induced by PIT does not totally rely on reactive oxygen species or the existence of singlet oxygen quenchers [9]. Furthermore, cytotoxicity induced by PIT is primarily within the cell membrane rather within the mitochondria as occurs with PDT.

While PIT results in rapid cellular necrosis, the overall volume of the tumor may not change for several days. This is because it takes at least several days for macrophages to enter, process and leave the treated tumor. Therefore, new methods, beyond size measurements, are needed to monitor the effects of PIT. Fluorescence proteins (FPs) are commonly used for visualizing cellular processes [10], [11]. While most apoptotic cell death results in preservation of the cell membrane and retention of the FP making fluorescence insensitive to cell death [12], [13], in PIT, the sudden rupture of cell membranes results in extrusion of cytoplasmic FPs and therefore, a relatively rapid readout of cell death. An advantage of FPs for *in vivo* imaging is that they do not require extrinsic injections of agents (such as luciferin in the case of bioluminescence) and can be monitored in real time [14]–[18]. Since they require gene transfection, they would likely only be useful in pre-clinical studies.

In this study, we examined the efficacy of PIT in a mouse model of disseminated peritoneal gastric cancer using in vivo GFP fluorescence imaging to monitor response.

## Materials and Methods

### Reagents

Water soluble, silicon-phthalocyanine derivative, IRDye 700DX NHS ester and IRDye 800 CW NHS ester were obtained from LI-COR Bioscience (Lincoln, NE, USA). Panitumumab, a fully humanized IgG_2_ mAb directed against EGFR, was purchased from Amgen (Thousand Oaks, CA, USA). Trastuzumab, 95% humanized IgG_1_ mAb directed against HER2, was purchased from Genentech (South San Francisco, CA, USA). All other chemicals were of reagent grade.

### Synthesis of IR700-conjugated trastuzumab or panitumumab, and IR800-conjugated trastuzumab

Conjugation of dyes with mAbs was performed according to a previous report [19]. In brief, panitumumab or trastuzumab (1 mg, 6.8 nmol) was incubated with IR700 NHS ester (60.2 µg, 30.8 nmol) or IRDye 800 CW NHS ester (35.9 µg, 30.8 nmol) in 0.1 mol/L Na_2_HPO_4_ (pH 8.6) at room temperature for 1 hr. The mixture was purified with a Sephadex G50 column (PD-10; GE Healthcare, Piscataway, NJ, USA). The protein concentration was determined with Coomassie Plus protein assay kit (Thermo Fisher Scientific Inc, Rockford, IL, USA) by measuring the absorption at 595 nm with spectroscopy (8453 Value System; Agilent Technologies, Santa Clara, CA, USA). The concentration of IR700 or IR800 was measured respectively by absorption at 689 nm or 774 nm with spectroscopy to confirm the number of fluorophore molecules conjugated to each mAb. The synthesis was controlled so that an average of four IR700 molecules and two IR800 molecules were bound to a single antibody. We performed SDS-PAGE as a quality control for each conjugate as previously reported [19]. We abbreviate IR700 conjugated to trastuzumab as tra-IR700, to panitumumab as pan-IR700 and IR800 conjugated to trastuzumab as tra-IR800.

### Cell culture

N87-GFP cells stably expressing GFP were purchased from ANTI CANCER (San Diego, CA, USA). High GFP expression was confirmed in the absence of a selection agent with 10 passages. To evaluate specific cell killing by PIT, 3T3 cells stably expressing DsRed (3T3/Dsred) were used as a negative control [5]. Cells were grown in RPMI 1640 (Life Technologies, Gaithersburg, MD, USA) supplemented with 10% fetal bovine serum and 1% penicillin/streptomycin (Life Technologies) in tissue culture flasks in a humidified incubator at 37°C at an atmosphere of 95% air and 5% carbon dioxide.

### Fluorescence microscopy

To detect the antigen-specific localization of IR700 conjugates and the change in cell morphology after PIT, fluorescence microscopy was performed (IX61 or IX81; Olympus America, Melville, NY, USA). Ten thousand cells were seeded on cover-glass-bottomed dishes and incubated for 24 hr. Tra-IR700 was then added to the culture medium (phenol red free) at 10 µg/mL and incubated at 37°C for 6 hr. The cells were then washed with PBS; Propidium Iodide (PI) (1∶2000) or Cytox Blue (1∶500) (Life Technologies) was added to the media 30 min before PIT to detect dead cells. The cells were then exposed to NIR light and serial images were obtained. One day after PIT, cells were washed and incubated with new medium after PIT (0.5 J/cm^2^) and PI was again added. To ensure that the same region was imaged markings were made on each culture dish to indicate where the imaging was acquired. The filter was set to detect IR700 fluorescence with a 590–650 nm excitation filter, and a 665–740 nm band pass emission filter. Analysis of the images was performed with ImageJ software (http://rsb.info.nih.gov/ij/).

### Flow Cytometry

Fluorescence from cells after incubation with pan-IR700 or tra-IR700 was measured using a flow cytometer (FACS Calibur, BD BioSciences, San Jose, CA, USA) and CellQuest software (BD BioSciences). Cells (1×10^5^) were incubated with each conjugate for 6 hr at 37°C. To validate the specific binding of the conjugated antibody, excess antibody (50 µg) was used to block 0.5 µg of dye-antibody conjugates [8].

### In vitro PIT

One hundred thousand cells were seeded into 24 well plates and incubated for 24 hr. Culture media was replaced with fresh culture media containing 10 µg/mL of tra-IR700 and the cells were incubated for 6 hr at 37°C. After washing with PBS, phenol red free culture medium was added. Then, cells were irradiated with NIR laser light at 685 to 695 nm wavelength (BWF5-690-8-600-0.37; B&W TEK INC., Newark, DE, USA). The actual power density of mW/cm^2^ was measured with an optical power meter (PM 100, Thorlabs, Newton, NJ, USA).

### Cytotoxicity assay

The cytotoxic effects of PIT with tra-IR700 were determined by flow cytometric PI staining, which detects compromised cell membranes. For the flow cytometric assay, cells were trypsinized 1 hr after treatment and washed with PBS. PI was added in the cell suspension (final 2 µg/mL) and incubated at room temperature for 30 min, followed by flow cytometry.

### Estimation of GFP fluorescence intensity in vitro

One hundred thousand cells were seeded on cover-glass-bottomed dishes and incubated for 12 hr. Tra-IR700 was then added to the culture medium (phenol red free) at 10 µg/mL and incubated at 37°C for 6 hr. The cells were washed with PBS and replaced with a new, phenol red free culture medium and the under side of the cover glass was marked (to determine the position of observation). After PIT, the cells were again incubated for 1 day. One day after PIT, the cells were again observed. The GFP intensity was evaluated with total pixels with the same threshold in the same field [20]. Analysis of the images was performed with ImageJ software (http://rsb.info.nih.gov/ij/).

Fluorescence from treated cells was also measured using a flow cytometer (FACS Calibur).

### Animal and tumor models

All *in vivo* procedures were conducted in compliance with the Guide for the Care and Use of Laboratory Animal Resources (1996), US National Research Council, and approved by the NIH Animal Care and Use Committee. Six- to eight-week-old female homozygote athymic nude mice were purchased from Charles River (NCI-Frederick). In order to evaluate only the direct tumor cell killing effect of *in vivo* PIT, immunocompromised nude mice were used. During procedures, mice were anesthetized with isoflurane. Ten million N87-GFP cells were injected subcutaneously in the right dorsum of the mice. In order to determine tumor volume, the greatest longitudinal diameter (length) and the greatest transverse diameter (width) were measured with an external caliper. Tumor volumes based on caliper measurements were calculated by the following formula; tumor volume  =  length × width^2^×0.5. Tumors reaching approximately 100 mm^3^ in volume were selected for the study. To measure GFP fluorescence after PIT, ten million N87-GFP cells were subcutaneously injected into both dorsi of the mice. For the disseminated peritoneal cancer mouse model, fifty million N87-GFP cells with PBS (total 300 µL) were injected into the peritoneal cavity.

### In vivo fluorescence imaging

*In vivo* fluorescence images were obtained with a Pearl Imager (LI-COR Bioscience) for detecting IR700/IR800 fluorescence, and a Maestro Imager (CRi, Woburn, MA, USA) for GFP. For GFP, a band-pass filter from 445 to 490 nm (excitation) and a long-pass blue filter over 515 nm (emission) were used. The tunable emission filter was automatically stepped in 10 nm increments from 500 to 600 nm for the green filter sets at constant exposure (500 msec). The spectral fluorescence images consist of autofluorescence spectra and the spectra from GFP (N87-GFP tumor), which were then unmixed, based on the characteristic spectral pattern of GFP, using Maestro software (CRi). Regions of interest (ROIs) were manually drawn either on the flank tumor or over the abdominal region as appropriate to the model and fluorescence intensity was measured [19].

### Characterization of disseminated peritoneal mouse model

Mice with either disseminated peritoneal cancer (at 6 weeks after cell implantation) or flank tumors (at 7 days after cell implantation) were injected intravenously with 100 µg of tra-IR700 and tra-IR800. One day after injection, serial images were performed with a Pearl Imager to detect IR700/IR800 fluorescence, and the Maestro for GFP. White light images of the mice were obtained with an iphone5 (Apple Inc., Cupertino, CA, USA).

### In vivo PIT

In order to evaluate the effect of PIT in the flank model, N87-GFP tumor-bearing mice were randomized into 4 groups of at least 10 animals per group as follows: (1) no treatment (control); (2) only NIR light exposure at 50 J/cm^2^ on day 1 and 100 J/cm^2^ on day 2; (3) 100 µg of tra-IR700 i.v., no NIR light exposure; (4) 100 µg of tra-IR700 i.v., NIR light was administered at 50 J/cm^2^ on day 1 after injection and 100 J/cm^2^ on day 2 after injection. These conditions were applied every week for up to 3 weeks. Mice were monitored daily, and fluorescence images were obtained beginning 1 day before PIT, and tumor volumes were measured three times a week until the tumor diameter reached 2 cm, whereupon the mice were euthanized with carbon dioxide. For fluorescence imaging, mice were injected with 100 µg of tra-IR700 or irradiated as follows: (1) NIR light was administered at 50 J/cm^2^ on day 1 after injection and 100 J/cm^2^ on day 2 to the right tumor (2) no NIR light was administered to the left tumor that served as the control. Controls included (1) only NIR light exposure at 50 J/cm^2^ on day 1 and 100 J/cm^2^ on day 2 to the right tumor; (2) no treatment for the left tumor.

To evaluate disseminated peritoneal cancer, mice were randomized into 4 groups of 5 animals per group for the following treatments: (1) no treatment (control); (2) only NIR light exposure at 50 J/cm^2^ on day 1 and 100 J/cm^2^ on day 2; (3) 100 µg of tra-IR700 i.v., no NIR light exposure; (4) 100 µg of tra-IR700 i.v., NIR light was administered at 50 J/cm^2^ on day 1 and 100 J/cm^2^ on day 2 after injection.

### Statistical Analysis

Data are expressed as means ± s.e.m. from a minimum of four experiments, unless otherwise indicated. Statistical analyses were carried out using a statistics program (GraphPad Prism; GraphPad Software, La Jolla, CA, USA). For multiple comparisons, a one-way analysis of variance (ANOVA) with post test (Kruskal-Wallis test with post-test) and Tukey’s test was used. The cumulative probability of survival, determined herein as the tumor diameter failing to reach 2 cm, was estimated in each group with the use of the Kaplan-Meier survival curve analysis, and the results were compared with the log-rank test and Wilcoxon test. Student’s *t* test was also used to compare the two *in vitro* study; p<0.05 was considered to indicate a statistically significant difference.

## Results

### Confirmation of expression profile of N87-GFP cells as a target for PIT

**Figure 1:**
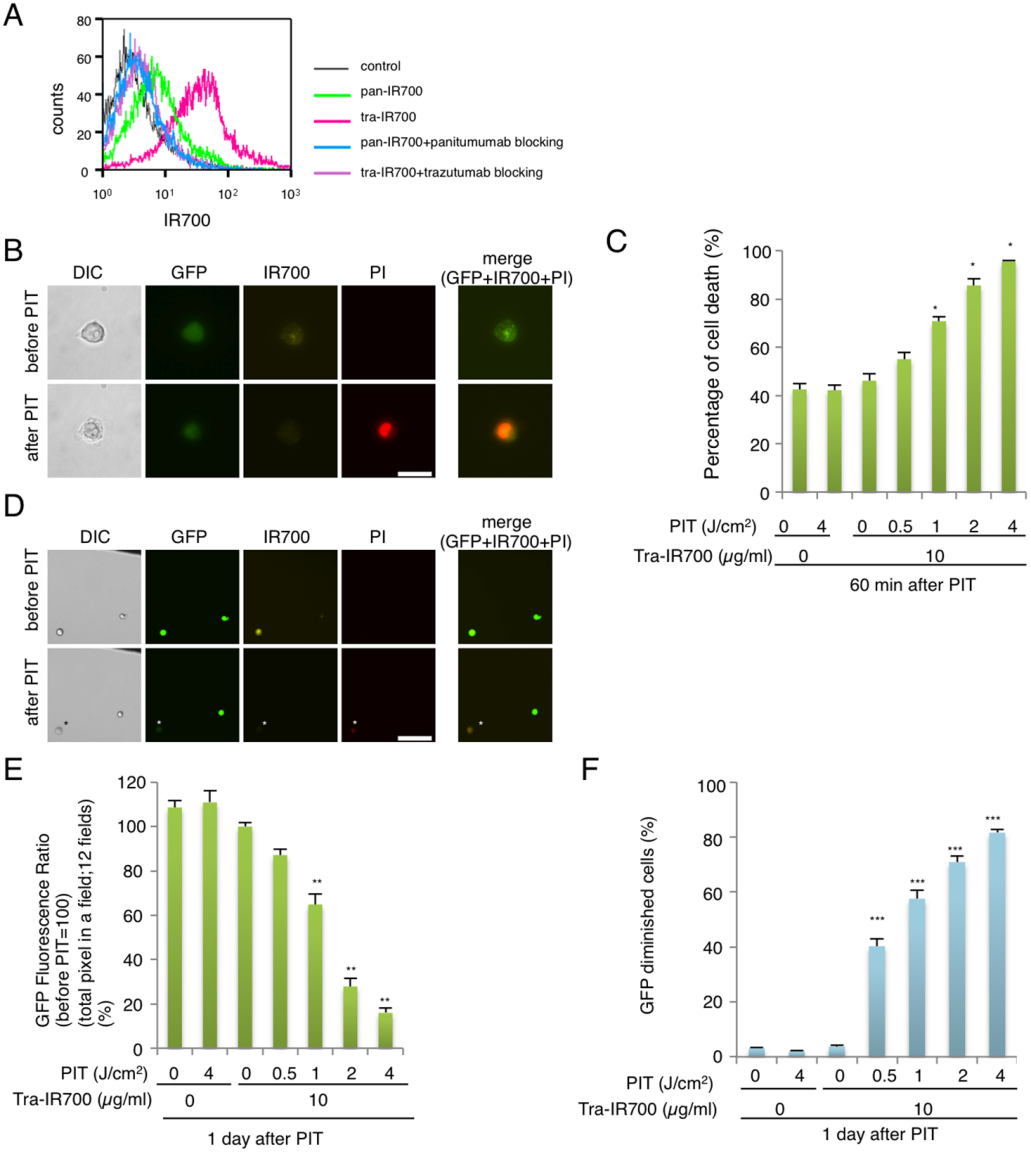
Confirmation of HER2 expression as a target for PIT in N87-GFP cells, and evaluation of *in vitro* PIT. (A) Expression of HER1 and HER2 in N87-GFP cells was examined with FACS. HER2 was overexpressed more than HER1. Specific binding was demonstrated by antibody blocking. (B) N87-GFP cells were incubated with tra-IR700 for 6 hr and observed by microscopy (Before and after irradiation of NIR light; 2 J/cm^2^). Necrotic cell death was observed upon excitation with NIR light (after 30 min). Bar = 25 µm. PI staining showed the membrane damage. (n = 4, *p<0.001, vs. untreated control, Student’s t test) (C) Membrane damage induced by PIT was measured with the dead cell count using PI staining, which increased in a manner dependent on the light dose. (D) N87-GFP cells were incubated with tra-IR700 for 6 hr and irradiated with NIR-light (0.5 J/cm^2^). GFP-fluorescence intensity decreased in dead cells but was unchanged in living cells at 1 day after PIT (*). Bar = 200 µm. The black line at right upper corner was the marker to determine the position of observation. (E) Diminishing GFP-fluorescence intensity at 1 day after PIT occurred in a manner dependent on the light dose (total pixel of GFP fluorescence in the same) (n = 12 fields) (**P<0.0001, vs. untreated control, Student’s t test). (F) Diminishing GFP fluorescence intensity induced by PIT, as measured by FACS, confirms a NIR-light dose-dependence. (n = 4, ***P<0.0001, vs. untreated control, Student’s t test).

We examined the fluorescence signals of pan-IR700 and tra-IR700 bound to N87-GFP cells by FACS. After 6 hr incubation with either pan-IR700 or tra-IR700, N87-GFP cells consistently showed higher brightness with tra-IR700 than pan-IR700 (Fig. 1A). These signals were almost entirely blocked by the addition of excess trastuzumab or panitumumab, suggesting specific binding and confirming the higher expression of HER2 than EGFR [21]. These data suggested that HER2 was the preferable target for PIT in N87-GFP cells due to its higher expression.

### Microscopy of in vitro PIT

Serial fluorescence microscopy of N87-GFP cells was performed before and after PIT. After exposure to NIR light (2 J/cm^2^) cellular swelling, bleb formation and rupture of the lysosome were observed, resulting in the extrusion of cytoplasm extracellularly (Fig. 1B). PI staining showed acute cytotoxic membrane damage caused by PIT. Most of these cellular changes were observed within 30 min of light exposure (Supporting Information video S1 and S2). No significant changes were detected in EGFR-negative 3T3 cells irradiated with NIR light, suggesting PIT induced no damage in non-target cells (Fig. S1).

### Evaluation of in vitro PIT effect

In order to quantitate the effect of *in vitro* PIT, we performed a cytotoxicity assay based on incorporation of PI, which demonstrated that cell death increased with increasing light dose (Fig. 1C). No significant cytotoxicity was detected with NIR light exposure or tra-IR700 alone.

### Evaluation with GFP fluorescence in vitro after PIT

One day after exposure to NIR light of 0.5 J/cm^2^, approximately 50% of cells showed acute cytotoxicity (Fig. 1C), and the GFP fluorescence intensity was greatly reduced in dead cells (stained positive with PI), while GFP fluorescence was preserved in surviving cells (Fig. 1D). These studies suggest that GFP was extruded from the cell after membrane rupture. In order to investigate the change in GFP fluorescence, we compared the total GFP pixels in the same field before and one day after PIT (Fig. S2). The GFP fluorescence ratio decreased directly with light dose, while no decrease was detected with NIR light exposure or tra-IR700 alone (Fig. 1E). These results were confirmed by FACS analysis (Fig. 1F and Fig. S3) and suggest that PIT leads to a decrease of GFP fluorescence following necrotic cell death at 1 day after PIT in a manner dependent on the light dose.

### In vivo PIT reduces tumor volume in flank xenograft model

**Figure 2:**
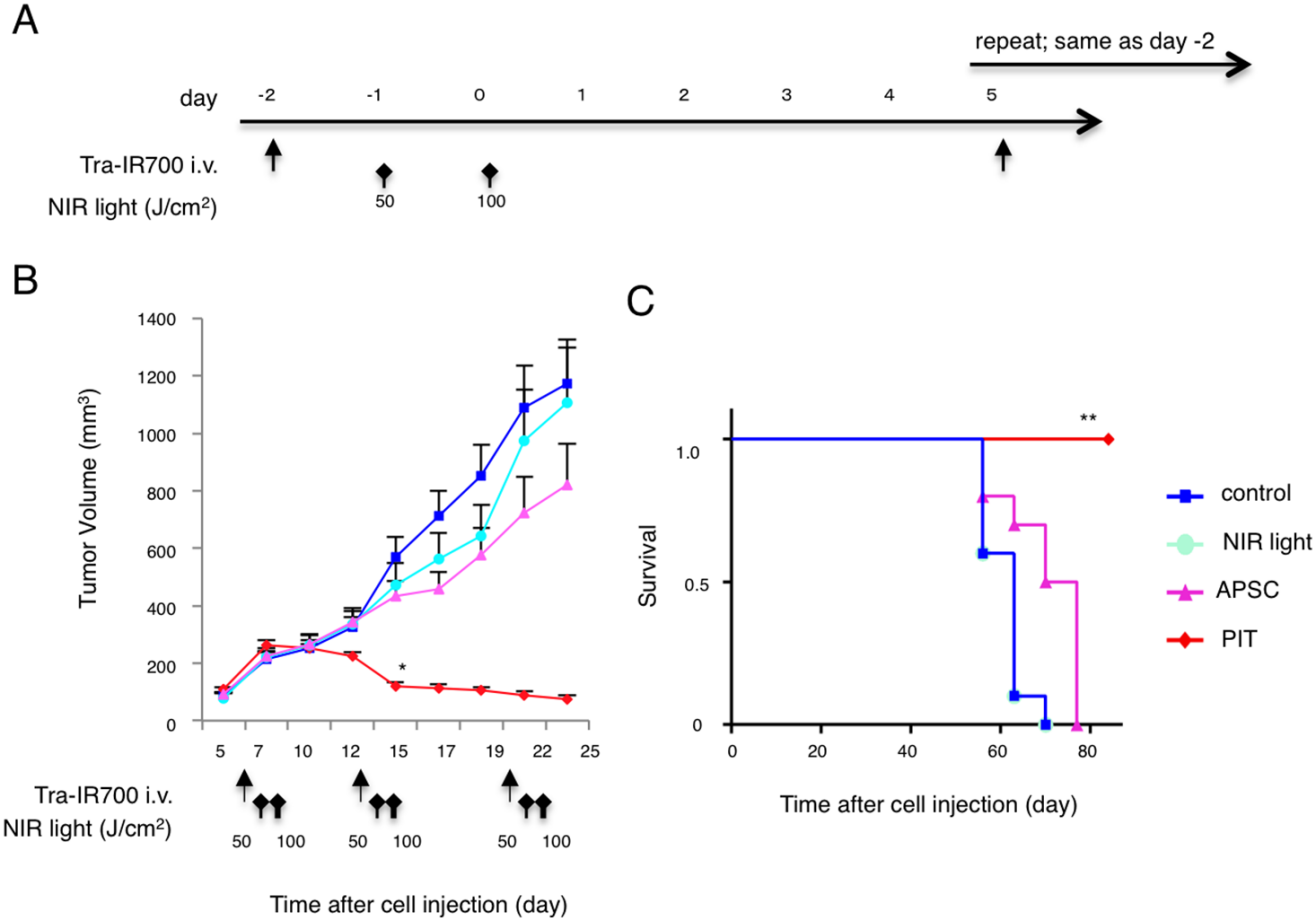
*In vivo* tumor growth inhibition by PIT in N87-GFP flank model. (A) PIT regimen. (B) PIT leads to N87-GFP flank tumor volume reduction (n = 10 mice in each treatment group; *p = 0.0456<0.05, vs. APSC), Tukey’s test with ANOVA). Treatment is indicated below the graph. (C) Repeated PIT leads to prolonged survival in N87-GFP tumor bearing mice (n = 10 mice in each treatment group, **P<0.0001), Long-rank test and Wilcoxon test). Complete tumor killing was achieved for 4 mice of 10 in the PIT group after re-treatment.

Next, we examined the effect of PIT *in vivo* on flank tumor bearing mice (Fig. 2A). PIT induced significant reductions in tumor volume (n = 10 in each group, *p = 0.0456<0.05, Tukey’s test). Four mice out of ten in PIT group were completely cured by the treatment (Fig. 2B). Survival was prolonged significantly in the PIT group compared with control group (n = 10 in each group, **p<0.0001, long-rank test and Wilcoxon test) (Fig. 2C). These data suggest that PIT caused significant tumor reduction and prolonged survival *in vivo*.

### GFP Fluorescence imaging after In vivo PIT in flank xenograft model

**Figure 3:**
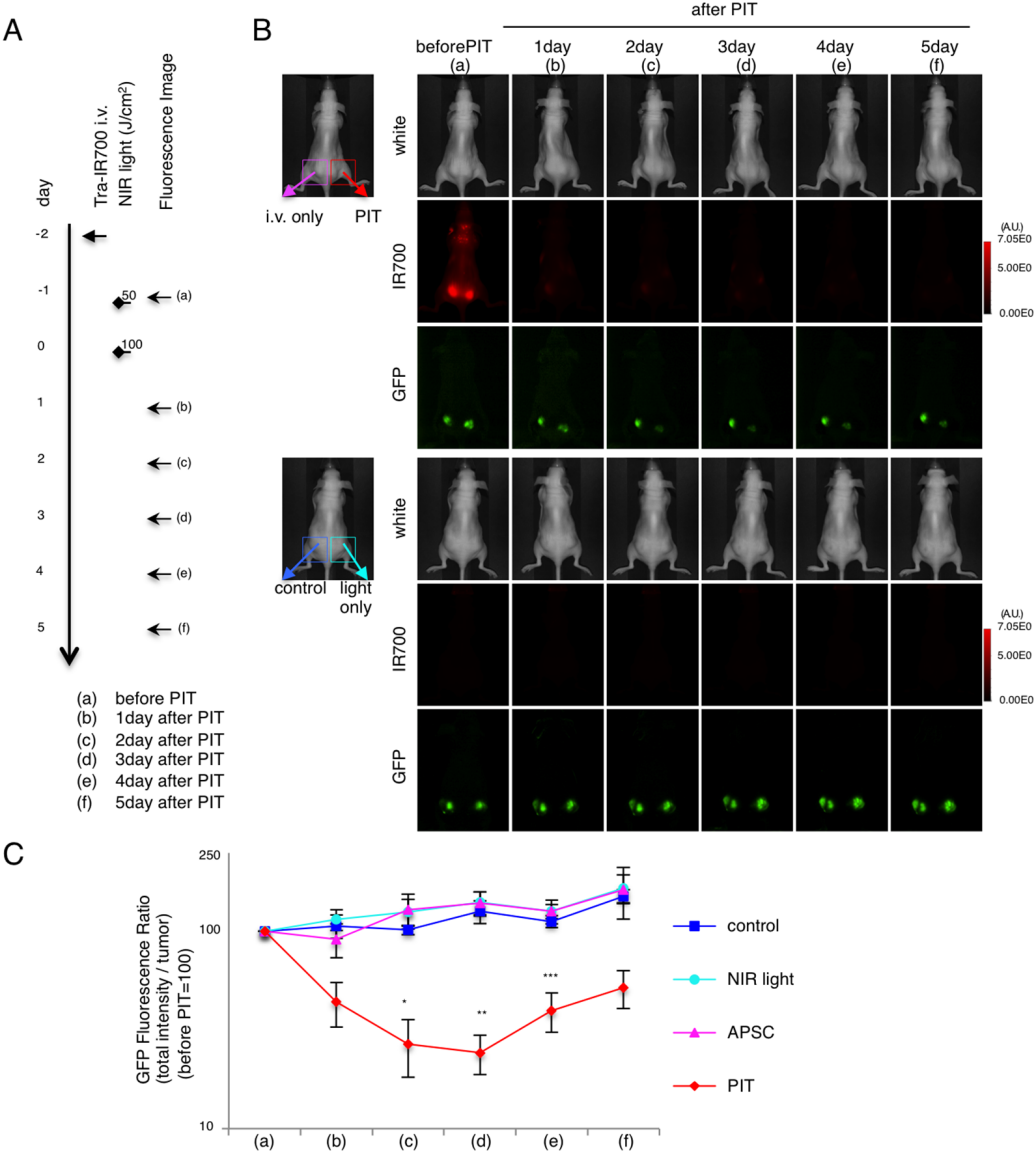
GFP fluorescence imaging of PIT *in vivo* in bilateral N87-GFP flank model. (A) PIT regimen. Fluorescence images were obtained at each time point as indicated. (B) *In vivo GFP* fluorescence real-time imaging of bilateral flank tumor bearing mice in response to PIT. The tumor treated by PIT showed decreasing GFP fluorescence after PIT. (C) Quantitative analysis of GFP fluorescence intensities (total intensity/tumor) in N87-GFP tumor bearing mice showed significantly decreased fluorescence between the control group and the PIT group (n = 5 mice in each group, (*p = 0.0118<0.05, vs. control) (*p = 0.0016<0.01, vs. NIR light) (*p = 0.0012<0.01, vs. APSC) (**p = 0.0010<0.01, vs. control) (**p = 0.0003<0.001, vs. NIR light) (**p = 0.0003<0.001, vs. APSC) (***p = 0.0049<0.01, vs. control) (***p = 0.0039<0.01, vs. NIR-light) (***p = 0.0012<0.01, vs. APSC), Tukey’s test with ANOVA).

In order to monitor *in vivo* PIT, real-time GFP imaging was performed in mice with N87-GFP bilateral flank tumors (Fig. 3A). GFP fluorescence demonstrated tumor burden and the response to PIT. GFP fluorescence was correlated with IR700 fluorescence, which rapidly decreased after PIT (Fig. 3C). Total fluorescence imaging (TFI) of GFP in untreated tumors (control) and in tumors receiving light only increased due to tumor growth. In tumors treated with PIT, TFI of GFP gradually decreased in the treated tumor, while the opposite tumor in the same mouse (i.v. only, no light) demonstrated increased GFP fluorescence (Fig. 3B).

Quantification of total GFP fluorescence intensity revealed that there was a significant decrease after PIT (n = 5 mice in each group, *p = 0.0118<0.05, **p = 0.0010<0.01, ***p = 0.0049<0.01, Tukey’s test with ANOVA) (Fig. 3C). Due to tumor re-growth, the GFP ratio increased again from day 4 after PIT, although it was lower than other control groups. The real-time GFP fluorescence imaging and its quantification enabled real-time comparison of the groups and showed a strong correlation between higher GFP fluorescence and tumor progression in each group.

**Figure 4:**
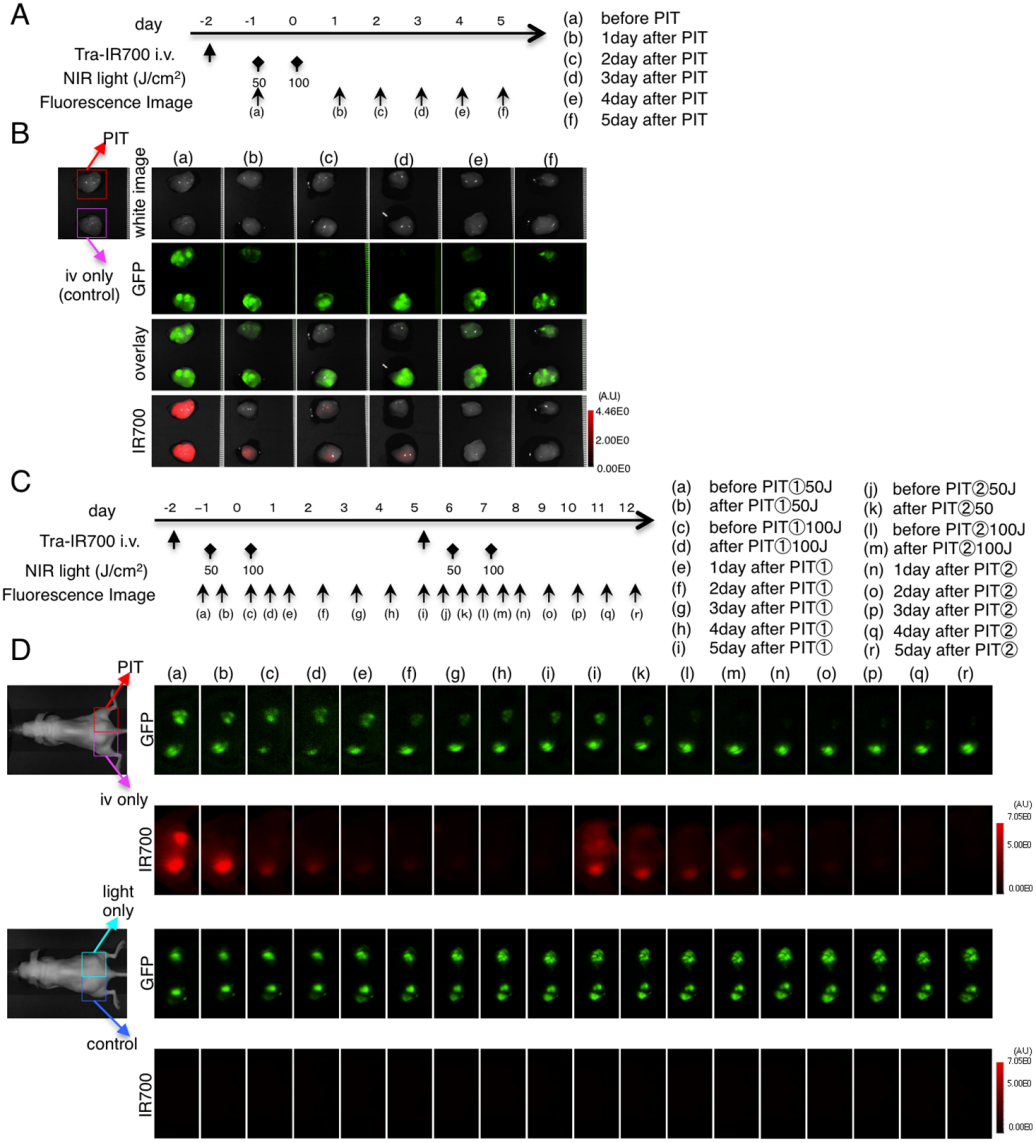
*Ex vivo* GFP fluorescence imaging after repeated PIT in bilateral N87-GFP flank model. (A) PIT regimen. Fluorescence images of *ex vivo* tumors were obtained at each time point as indicated. (B) *Ex vivo* fluorescence imaging of N87-GFP tumor in response to PIT. Fluorescence changes are seen with both GFP and IR700 fluorescence in response to PIT. (C) Fluorescence images were obtained at each time point as indicated. (D) *In vivo* fluorescence real-time imaging of bilateral flank tumor bearing mice in response to repeated PIT. GFP-fluorescence intensity of the tumor was almost eliminated after the second PIT.

To confirm this effect, *ex vivo* analysis was performed (Fig. 4A). The PIT effect was confirmed with a decrease in IR700 fluorescence (Fig. 4B). Under this condition, GFP intensity of *ex vivo* tumors decreased after PIT (Fig. 4B). These data suggest that real-time GFP live imaging is consistent with *ex vivo* imaging.

When PIT was repeated weekly, GFP fluorescence activity eventually disappeared (Fig. 4C and D), indicating complete killing of the tumor.

### Characterization of the disseminated peritoneal model with fluorescence imaging

**Figure 5:**
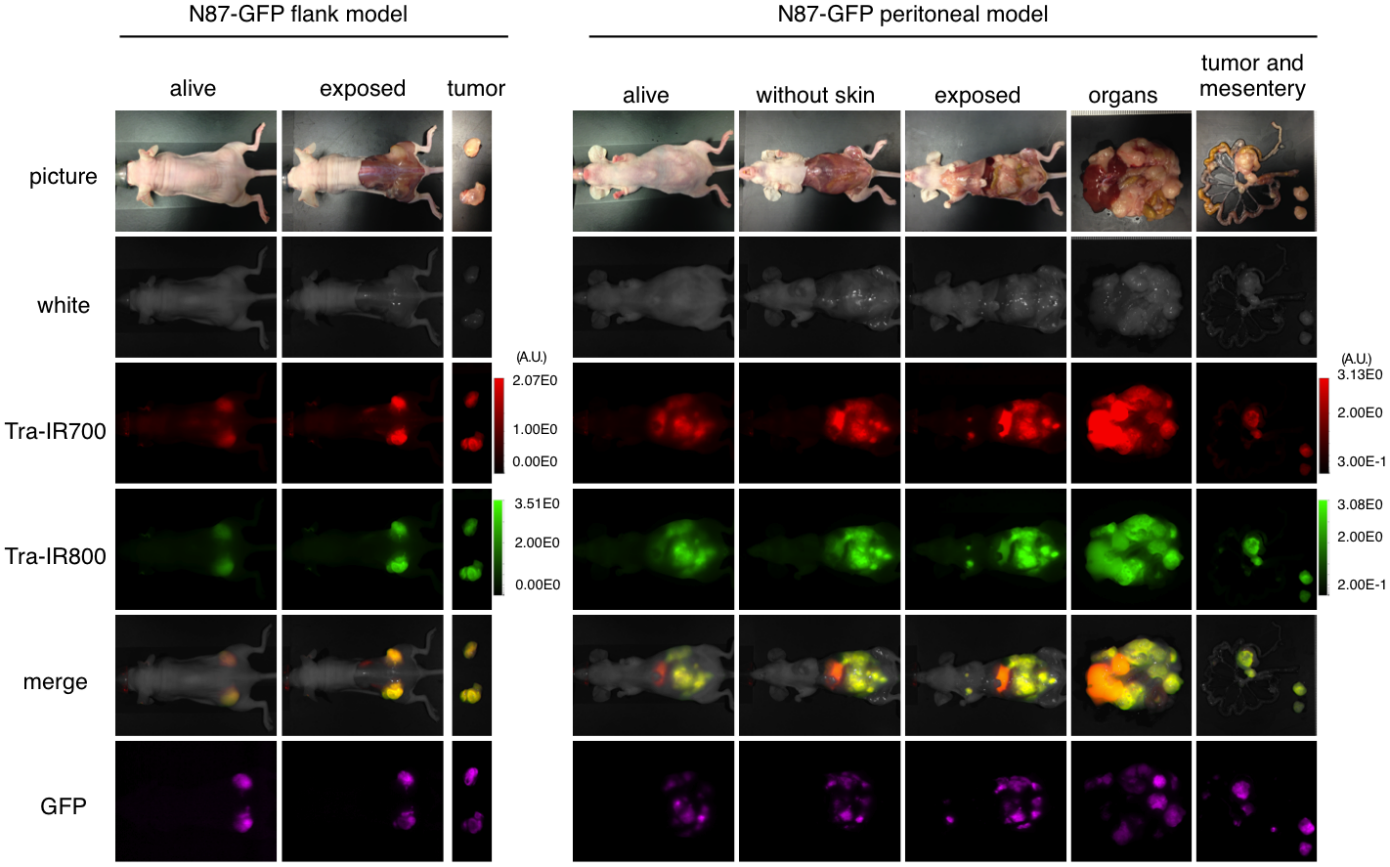
GFP fluorescence imaging of N87-GFP flank and disseminated peritoneal model. *In vivo* GFP fluorescence imaging of a N87-GFP flank tumor and disseminated peritoneal model. Colocalization of tra-IR700 and tra-IR800 was confirmed in the peritoneal disseminated tumor with fluorescence imaging. Intravenous injection of the agent can reach disseminated tumors in the peritoneal cavity. To avoid auto-fluorescence in the intestine, tra-IR800 was used as well as tra-IR700.

In order to determine the natural history of the disseminated peritoneal model, serial fluorescence imaging was performed. The implanted tumors demonstrated high fluorescence signal with IR700, IR800, and GFP, all of which co-localized with each other (IR800 was used to avoid intestinal autofluorescence) (Fig. 5). These data suggest that this model of disseminated peritoneal cancer can be monitored in live mice using GFP fluorescence.

### GFP Fluorescence imaging after In vivo PIT in disseminated peritoneal model

**Figure 6:**
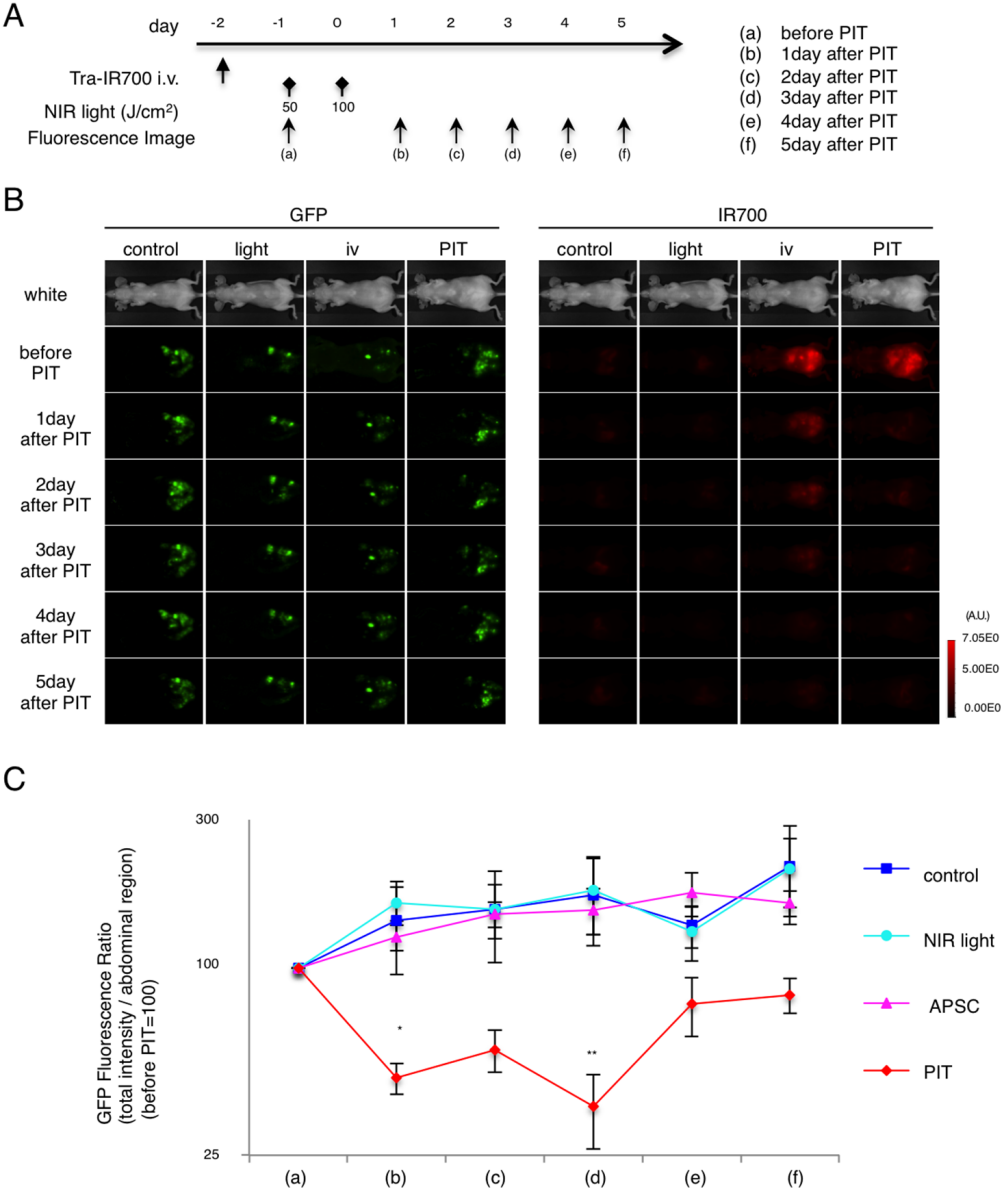
Evaluation of PIT effects in a disseminated peritoneal model. (A) PIT regimen. Real-time GFP fluorescence imaging was obtained at each time point indicated. (B) *In vivo* real-time fluorescence imaging in response to PIT. (C) Quantitative analysis of GFP fluorescence intensities showed significant decreases by day 3 after PIT between control group and PIT group (n = 5 mice in each group, (*p = 0.0295<0.05 vs. NIR light) (**p = 0.0489<0.05 vs. control) (**p = 0.0355<0.05 vs. APSC), Kruskal-Wallis test with post-test).

Real-time GFP fluorescence imaging was performed in the disseminated peritoneal model before and after PIT (Fig. 6A). IR700 fluorescence decreased after PIT (Fig. S4). GFP TFI gradually increased in the non-PIT groups due to tumor growth. In the PIT treated group, GFP TFI decreased from 1 day to 3 day after PIT (Fig. 6B). Quantification of total GFP fluorescence intensity revealed a significant decrease after PIT (n = 5 mice in each group, *p = 0.0295<0.05, **p = 0.0355<0.05, Kruskal-Wallis test with post-test) (Fig. 6C). Due to the tumor re-growth, the GFP ratio increased again from 4 day after PIT, although it maintained lower than other groups, as observed with the flank tumor model. Thus, PIT caused significant targeted tumor killing effect in both the flank and disseminated peritoneal tumor models and this could be monitored with GFP TFI.

## Discussion

This study demonstrates that the effect of PIT can be monitored with GFP fluorescence imaging. Unlike apoptosis [12], [13], in which GFP and related fluorescent proteins become brighter due to the decreasing cytoplasmic volume, during necrotic cell death with PIT, membrane damage leads to release of cytoplasmic contents including GFP. This unique characteristic of GFP and related fluorescent proteins makes them useful biomarkers for PIT.

*In vivo* GFP fluorescence imaging enables the full process of tumorigenesis, treatment, regression, metastasis, or recurrence, to be detected in the same animal without invasive procedures [10], [11]. By employing cells expressing cytoplasmic GFP, the antitumor effects induced by PIT could be readily monitored due to extrusion of GFP from treated cells.

The concept of using targeted light therapy is over three decades old [22]. Due to the hydrophobicity of traditional photodynamic therapy (PDT) sensitizers, the pharmacokinetics of antibody conjugated PDT agents is highly limited in its ability to deliver conjugates to the target. Therefore, PDT sensitizers targeted with antibodies has only been successful in models where the conjugate was injected directly into the tumor or peritoneum [23]. In order to deliver an antibody-photosensitizer conjugate (APSC) systemically, the photosensitizer should be preferentially hydrophilic. The hydrophilic phthalocyanine-based photosensitizer, IR700DX when conjugated to an antibody becomes an APC that can be administered intravenously. This form of therapy, termed PIT, differs from traditional PDT not only in the hydrophilicity of the photosensitizer, but also in its reliance on NIR light to activate the photosensitizer. NIR light has better tissue penetration than lower wavelength light used in PDT. This new generation of APCs demonstrates similar intravenous pharmacokinetics to naked antibodies, resulting in highly targeted tumor accumulation with minimal non target binding and can be applied to a broad spectrum of antibodies, allowing many potential applications throughout the body.

Despite cytotoxic chemotherapy, the outcome for advanced stage gastric cancer remains poor. Because, the majority of patients with peritoneal metastases present with insidious symptoms such as poor appetite, weight loss, bloating sensation, pain or anemia diagnosis is often delayed and local therapies are no longer feasible [24]. PIT is a promising method of treating disseminated peritoneal cancer. In order to perform effective PIT, the APSC should be delivered systemically and the light should be selectively applied to the peritoneum. Under these conditions there is sufficient delivery of APSCs to result in reduction of disseminated tumors after PIT. This process can be monitored with real time *in vivo* GFP fluorescence imaging.

There are several limitations to this study. It should be noted that not all gastric cancers express HER2 and therefore, tra-IR700 may not be uniformly effective in disseminated gastric cancer. Indeed, it may be necessary to use of a cocktail of APSCs to effectively cover most of the expression profiles in a given tumor [25]. Another caveat in this study is that light could be administered transcutaneously in small mice but this is not possible in humans. In order to effectively treat humans with PIT, it will be necessary to apply light laparoscopically to prevent absorption by the skin. Another limitation is that our model was only evaluated in immunocompromised mice. The immunological reaction of PIT will be evaluated in the future in immunocompetent mice. Finally, in the near term GFP fluorescence imaging will be limited to animal studies, as it requires transfection of the tumor with an endogenous protein. Nevertheless, this is a useful readout for preclinical studies.

## Conclusions

PIT is effective against HER2-positive gastric cancer peritoneal carcinomatosis in a mouse model and GFP fluorescence imaging enables monitoring of PIT effects.

## Supporting Information

Figure S1 **Specific targeted necrotic cell death was observed after PIT** ***in vitro.*** N87-GFP cells were co-cultured with 3T3/DsRed (non-HER expressing) cells. They were treated with tra-IR700 and observed (before and after irradiation of NIR light). Targeted specific necrotic cell death was observed upon excitation with NIR light (after 30 min). No damage was demonstrated in 3T3/DsRed cells. * 3T3/DsRed cells, Bar = 25 µm.
(TIFF)

Figure S2 **Diminishing of GFP-fluorescence at 1 day after PIT was observed** ***in vitro.*** Diminishing GFP-fluorescence intensity at 1 day after PIT was observed to be in a manner dependent on the light dose. Bar = 100 µm. The black line at the edge was the marker to determine the position of observation.
(TIFF)

Figure S3 **Decreases in GFP-fluorescence at 1 day after PIT evaluated by flow cytometry.** Diminishing GFP-fluorescence intensity at 1 day after PIT was in a manner dependent on the light dose by flow cytometric analysis.
(TIFF)

Figure S4 *In vivo* fluorescence imaging in response to repeated PIT in the peritoneal disseminated mice model. *In vivo* fluorescence imaging in response to repeated PIT. IR700 fluorescence was decreased in response to PIT.
(TIFF)

Video S1 **Time-lapse DIC images of a N87-GFP cell treated by PIT.** Time-lapse sequential DIC images (video) of a N87-GFP cell shows morphologic changes of the cell after NIR light irradiation in cells treated with tra-IR700 (for 25 min observation in total). Flashing light is NIR light irradiation (2 J/cm^2^).
(AVI)

Video S2 **Time-lapse PI fluorescence images of a N87-GFP cell treated by PIT.** Time-lapse sequential PI fluorescence images (video) shows rapid membrane damage of a N87-GFP cell after NIR light irradiation in cells treated with tra-IR700 (for 25 min observation in total). Flashing light is NIR light irradiation (2 J/cm^2^).
(AVI)
