# Supplementary figures and images for: Photoimmunotherapy of Gastric Cancer Peritoneal Carcinomatosis in a Mouse Model
Source: PLoS One. 2014 Nov 17;9(11):e113276. doi: 10.1371/journal.pone.0113276 (PMC4234664; doi:10.1371/journal.pone.0113276)

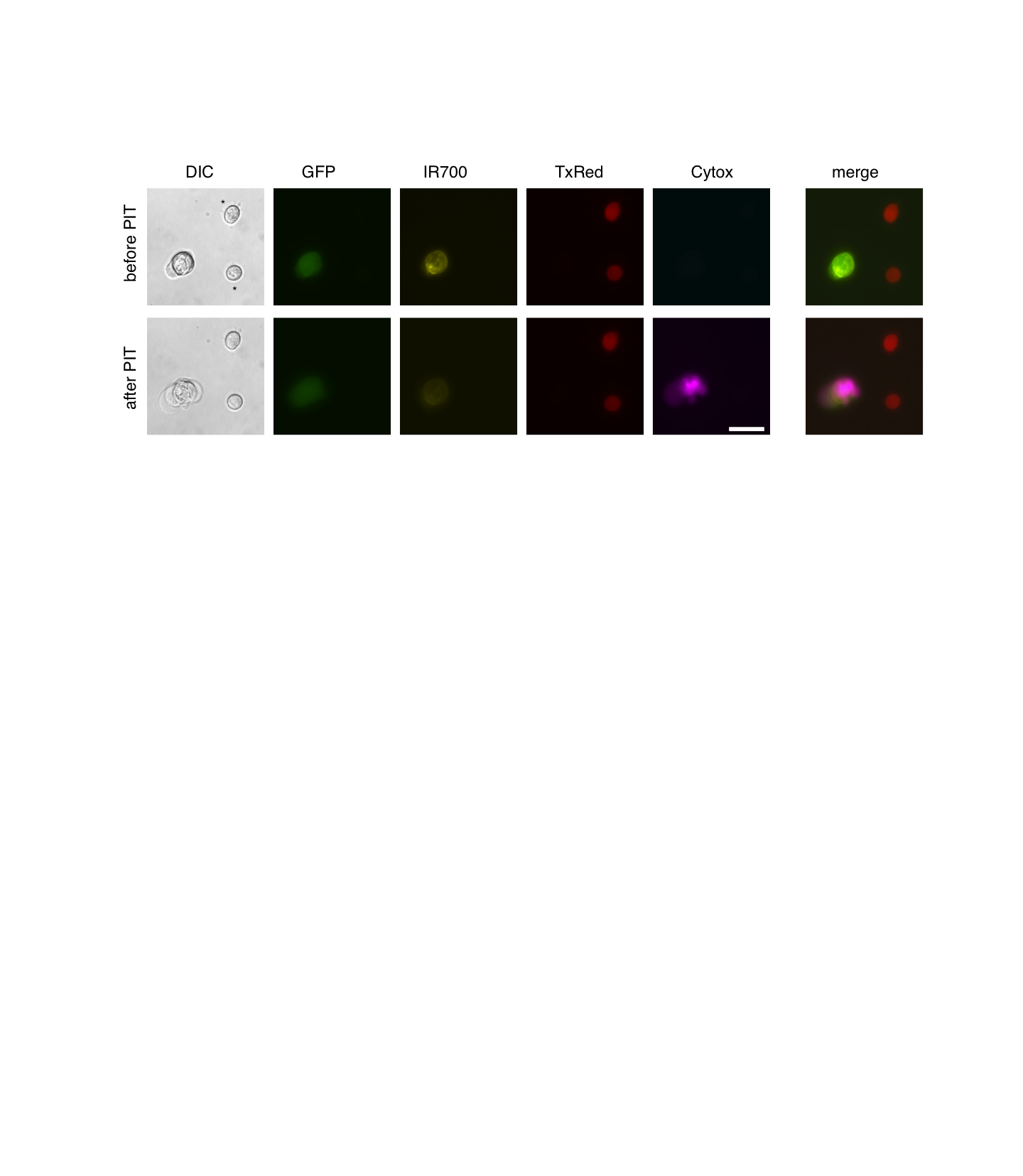

Supplement: Figure S1 — Specific targeted necrotic cell death was observed after PIT in vitro. N87-GFP cells were co-cultured with 3T3/DsRed (non-HER expressing) cells. They were treated with tra-IR700 and observed (before and after irradiation of NIR light). Targeted specific necrotic cell death was observed upon excitation with NIR light (after 30 min). No damage was demonstrated in 3T3/DsRed cells. * 3T3/DsRed cells, Bar = 25 µm. (TIFF) [file pone.0113276.s001.tiff]

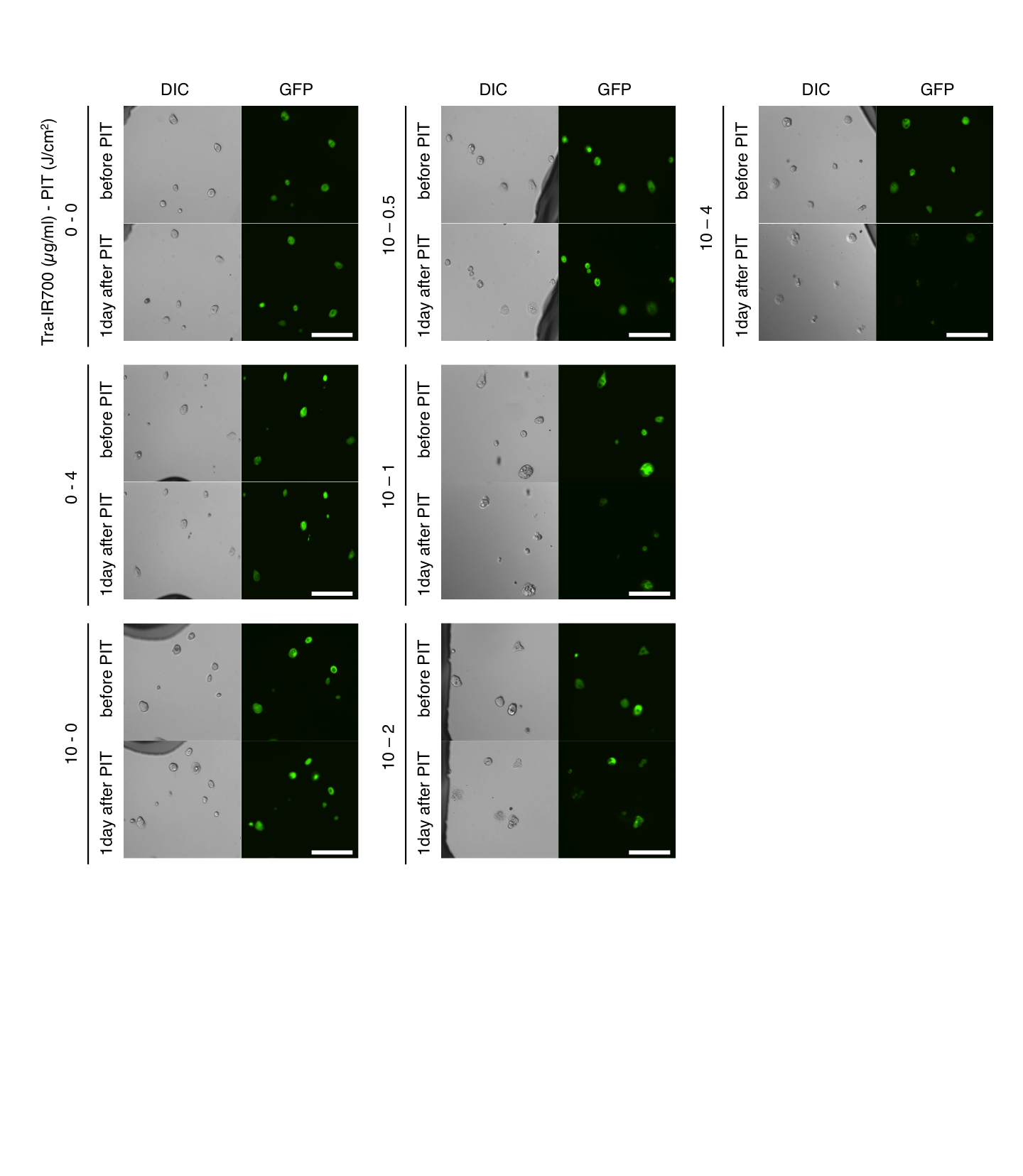

Supplement: Figure S2 — Diminishing of GFP-fluorescence at 1 day after PIT was observed in vitro. Diminishing GFP-fluorescence intensity at 1 day after PIT was observed to be in a manner dependent on the light dose. Bar = 100 µm. The black line at the edge was the marker to determine the position of observation. (TIFF) [file pone.0113276.s002.tiff]

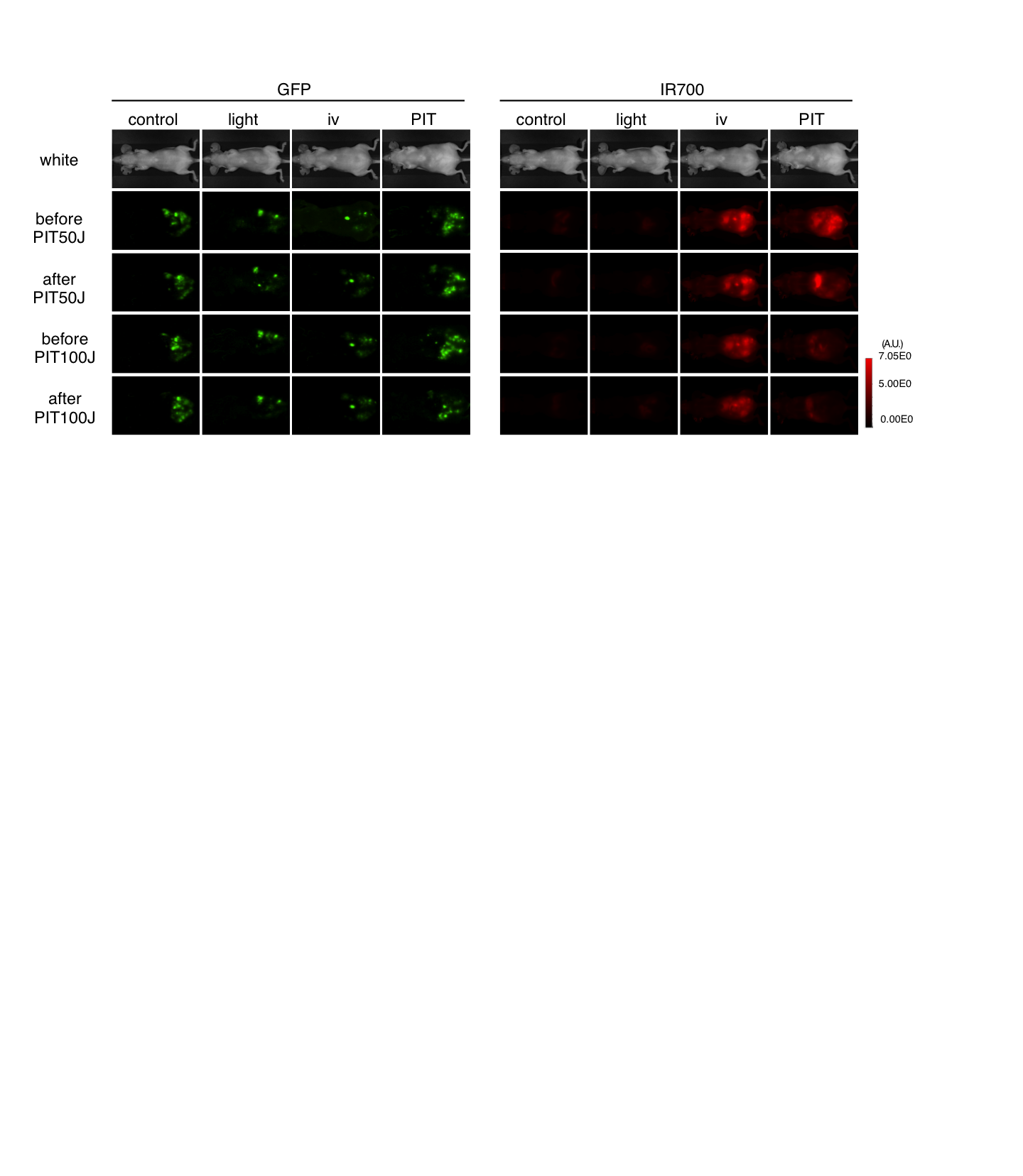

Supplement: Figure S4 — In vivo fluorescence imaging in response to repeated PIT in the peritoneal disseminated mice model. In vivo fluorescence imaging in response to repeated PIT. IR700 fluorescence was decreased in response to PIT. (TIFF) [file pone.0113276.s004.tiff]
